# Spontaneous Iliopsoas Hematoma following Microvascular Free Tissue Transfer

**DOI:** 10.1155/2017/7631673

**Published:** 2017-03-26

**Authors:** Jeffrey D. Markey, A. Sean Alemi, Margaret L. Naunheim, Daniel L. Faden, Chase M. Heaton, Rahul Seth

**Affiliations:** Department of Otolaryngology-Head and Neck Surgery, University of California, San Francisco, San Francisco, CA, USA

## Abstract

Spontaneous hematoma within the iliopsoas muscle (SIH) is a rare complication most commonly seen in coagulopathic patients. Often, patients undergoing microvascular free tissue transfer are anticoagulated for anastomotic patency. Here we describe two cases of postoperative SIH following contralateral anterolateral thigh (ALT) free tissue transfer for reconstruction of oncologic head and neck defects. Both patients described hip pain after mobilization and had a corresponding acute blood loss anemia. Diagnosis of SIH was confirmed by CT and both patients were managed conservatively. Given that anticoagulation is a common practice following head and neck free tissue transfer, surgeons should be aware of this potential complication.

## 1. Introduction

Spontaneous hematoma within the iliopsoas muscle (SIH) is a rare complication most commonly seen in coagulopathic patients. Active pharmacologic anticoagulation for venous thromboembolic events and acute coronary disease, perioperative anticoagulation, and congenital conditions such as hemophilia have been associated with coagulopathy leading to SIH [1–6]. Patients undergoing resection of head and neck tumors with free tissue transfer reconstruction are often anticoagulated to prevent formation of anastomotic thrombus. The optimal anticoagulation protocol is not known and remains heavily debated in the literature. Despite the common practice of anticoagulation after head and neck free tissue transfer, we are unaware of any reported cases of SIH in this patient population. Here, we describe two cases of patients undergoing anterolateral thigh (ALT) free tissue transfer complicated by postoperative contralateral SIH.

## 2. Case Reports

### 2.1. Case 1

**Figure 1:**
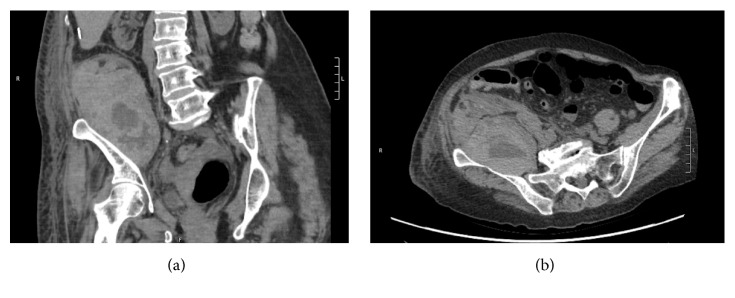
Computed tomography images without contrast in coronal plane (a) and axial plane (b) demonstrating right-sided iliopsoas hematoma as presented in case 1.

A 72-year-old female with T2N0 left buccal squamous cell carcinoma underwent tracheotomy, wide local excision of left buccal lesion, left maxillectomy, left neck dissection levels I–IV, and left ALT free flap reconstruction. Her medical history included stage V chronic kidney disease resulting in anemia, hypertension, and coronary artery disease with prior stent placement requiring chronic anticoagulation with clopidogrel. Clopidogrel was held five days prior to surgery. The surgery was uncomplicated. Aspirin 300 mg PR was administered immediately postoperatively. Thereafter, she received 81 mg aspirin per feeding tube daily and 5,000 units heparin subcutaneously TID. The patient remained hemodynamically stable and normotensive in the postoperative period. She was mobilized to chair on postoperative day (POD) 2 and was ambulating with assistance on POD 3. On POD 5, the patient's hemoglobin levels decreased from 9.5 g/dL to 7.0 g/dL. During this time, she also developed leukocytosis to 21.5 × 10^9^/L and complained of hip pain contralateral to the ALT donor site, affecting her ability to perform weight-bearing activity. Orthopedic surgery was consulted and noted lower extremity weakness with hip extension for which a CT without contrast was ordered. Imaging revealed a 6.9 × 9.3 × 14.3 cm hematoma within the distal iliopsoas muscle without clear evidence of infection (Figures 1(a) and 1(b)). Subcutaneous heparin was stopped and the patient received a blood transfusion with an appropriate increase in hemoglobin level. The patient was discharged home and was ambulating without assistance on POD 15.

### 2.2. Case 2

**Figure 2:**
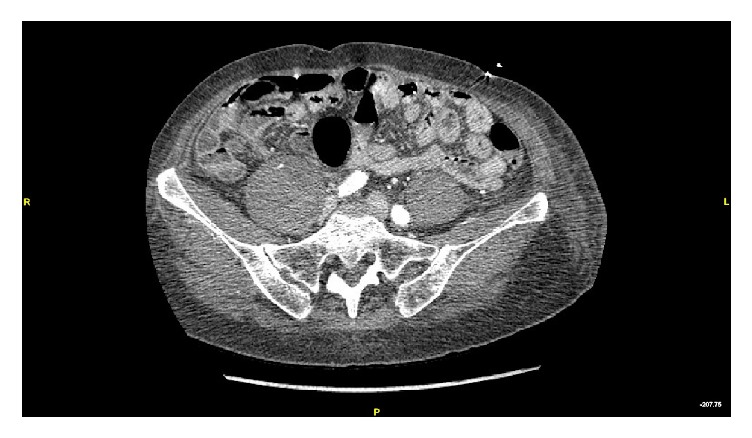
Computed tomography image with contrast in axial plane demonstrating right-sided iliopsoas hematomas as presented in case 2.

A 77-year-old female with a past medical history of hypertension and T3N2cM0 SCC of the left buccal mucosa underwent full thickness buccal excision, left selective neck dissection of levels I–III, and left ALT for reconstruction. Surgery was uncomplicated. She was started on a heparin drip titrated to a PTT goal of 65 seconds and given aspirin 325 mg daily postoperatively according to reconstructive surgeon's preference. She was mobilized to a chair on POD 2 and began ambulating with assistance on POD 3. On POD 4, the patient developed tachycardia to greater than 150 beats/minute. Complete blood count demonstrated a drop in hemoglobin from 9.5 g/dL to 7.1 g/dL by POD 8. She was transfused with subsequent appropriate correction of anemia. On POD 9, the patient complained of right lower quadrant abdominal pain. Physical exam at that time demonstrated pain with active flexion and passive extension of the right hip and decreased tolerance for weight-bearing activity on that side. The orthopedic surgery service was consulted and recommended conservative management. The patient's clinical condition did not improve, so a contrast-enhanced CT of the abdomen and pelvis was obtained on POD 10 which demonstrated a psoas muscle hematoma (Figure 2). Anticoagulation was held and hemoglobin levels remained stable. The patient was ambulating with assistance and was discharged to a skilled nursing facility on POD 12.

## 3. Discussion

SIH is most commonly seen in patients with type A or type B hemophilia but has also been noted in anticoagulated patients and even in patients with no bleeding diathesis [1, 2, 7]. Patients undergoing head and neck free flap reconstruction are often anticoagulated postoperatively. Anticoagulation is associated with postoperative hematoma formation, although almost always at a surgical site [8].

The classic presentation of SIH is sudden to subacute paraspinal, groin, abdominal, and/or hip pain, accompanied by a >2 g/dL drop in hemoglobin level [3]. Physical exam can reveal the “psoas sign,” pain with passive extension of the hip correlating to stretching of the iliopsoas. Femoral nerve compression can also occur, which manifests clinically as paresthesia of the anterior thigh or anteromedial calf with later signs including lower extremity extension, flexion, or abduction weakness [2]. The femoral nerve traverses through the psoas muscle fibers, descends via the iliopsoas groove, and passes deep to the medial inguinal ligament, rendering it particularly susceptible to compression; thus, these symptoms require expedited diagnostic work-up [2].

CT is a fast, highly sensitive, and commonly used imaging modality to diagnose SIH. Ultrasound has also been described to have similar diagnostic accuracy [9]. The most appropriate treatment option for SIH depends on the clinical scenario. Conservative management with observation, bedrest, and correction of anemia is often sufficient [2–4]. Nonconservative interventional options include transarterial embolization (TAE), ultrasound guided percutaneous aspiration, and/or surgical decompression for more severe cases [4, 5].

Theories as to the etiology of SIH include small tears in the muscle fibers, small-vessel arteriosclerosis, heparin-induced immune microangiopathy, and unrecognized minor trauma [4, 6]. SIH can occur upon initiation of mobilization after prolonged periods of bedrest, spontaneously in coagulopathic sedated patients, or even in noncoagulopathic patients merely performing repetitive movements [6, 7]. The relative risk incurred regarding specific modalities of anticoagulation is not known. In the present report, different types of anticoagulation and antiplatelet therapy were administered in either case. Any quantifiable causative role of the therapy regarding the development of SIH remains unclear.

Lee et al. (2015) describe a patient receiving anticoagulation following mechanical aortic valve replacement developing a unilateral SIH, undergoing TAE, and then subsequently developing a contralateral SIH noted on repeat imaging [4]. The authors suggest that, by favoring the unaffected contralateral leg to relieve discomfort in the initial SIH-affected limb, the patient inadvertently suffered contralateral iliopsoas trauma, resulting in a second SIH. We favor a similar explanation for the development of SIH following ALT free flap surgery within the iliopsoas muscle contralateral to the donor site. In both scenarios, the patients developed symptoms after initiation of mobilization.

One of the advantages of the ALT flap is the low donor site morbidity, making it favored at our institution for many defects. Although rare, SIH should be considered in patients undergoing ALT surgery who develop contralateral hip pain along with unaccountable drop in hemoglobin level.

## 4. Conclusion

SIH is a rare complication which occurs most often in anticoagulated or coagulopathic patients. Patients undergoing free tissue transfer for reconstruction of head and neck defects are frequently anticoagulated postoperatively and are at risk for bleeding complications. While most commonly this occurs at the donor or recipient sites, here we describe two cases of SIH following contralateral ALT surgery. We hypothesize that this is due to favored weight-bearing on the contralateral leg at the time of postoperative mobilization. SIH should be considered in the differential diagnosis for a patient who develops anemia in the context of lower extremity pain, paresthesia, or weakness following ALT surgery.
